# The Role Ionic Liquid [BMIM][PF_6_] in One-Pot Synthesis of Tetrahydropyran Rings through Tandem Barbier–Prins Reaction

**DOI:** 10.3390/molecules24112084

**Published:** 2019-05-31

**Authors:** Poliane K. Batista, João Marcos G. de O. Ferreira, Fabio P. L. Silva, Mario L. A. A Vasconcellos, Juliana A. Vale

**Affiliations:** Departamento de Química, Universidade Federal da Paraíba, Cidade Universitária, 58051-900 João Pessoa-PB, Brazil; polianekarenine@gmail.com (P.K.B.); profjoaomarcos@hotmail.com (J.M.G.d.O.F.); pedrosalinssilva@gmail.com (F.P.L.S.); mlaav00@gmail.com (M.L.A.A.V.)

**Keywords:** ionic liquids, tetrahydropyran, Barbier–Prins Reaction, catalysis, [BMIM][PF_6_]

## Abstract

Tetrahydropyran (THP) rings are common in several natural products, therefore, various strategies are being developed to synthesize these rings. The present work described the study of a one-pot synthesis of 2,4,6-trisubstituted tetrahydropyran compounds promoted by the ionic liquid 1-butyl-3-methylimidazolium hexafluorophosphate [BMIM][PF_6_] through a Barbier–Prins reaction between allyl bromide and aldehydes. The use of [BMIM][PF_6_] gave Prins products from several aldehydes in good yields and reaction times. We also found that the anion, PF_6_-, accelerates the Barbier reaction when used alone, and the excess SnBr_2_ from the reaction conditions of the Barbier reaction leads to the formation of the THP rings, thus acting as a catalyst for Prins cyclization. Additionally, we demonstrate that ionic liquid can be recovered and reused five times in the preparation of 4-bromo-tetrahydro-2,6-diphenyl-2H-pyran without significant yield loss.

## 1. Introduction

A significant number of biologically important natural products contain tetrahydropyran (THP) rings, such as neurotoxin brevetoxin B [1] and the antibiotics monensin and 17-deoxyroflamycoin [2]. THP’s rings present interesting biological, pharmaceutical, antimicrobial [3], antifungal [4], antitumor [5], antiviral [6], analgesic [7], anti-inflammatory [8], and antidiabetic [9] properties. Thus, the THP skeleton has become an attractive object of study in the field of organic synthesis.

As reactions of hetero-Diels–Alder [10], iodolactonization reaction [11], selenium etherification of unsaturated alcohols [12], 6-endo cyclization of vinyl epoxides [13], reductive etherification [14], ring closing metathesis [15], Takeda olefination [16], SmI_2_-mediated radical cyclization [17], Iterative epoxide opening cyclization [18], and Prins cyclization reaction are some synthesis methodologies developed aiming at the formation of tetrahydropyran rings.

**Scheme 1:**
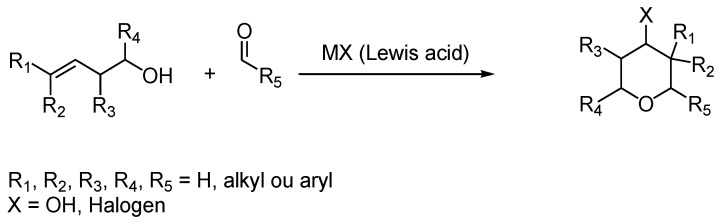
Lewis acid mediated Prins cyclization reaction.

The Prins cyclization is a synthetic methodology developed with the objective of forming THP rings that commonly occur between homoallylic alcohols (or ethers) and aldehydes (or acetals), primarily mediated by a Lewis acid (AlCl_3_ [19], SnCl_4_ [20], SnBr_4_ [21], InCl_3_ [22], among others) (Scheme 1). A more viable alternative for forming THPs rings is a one-pot synthesis under traditional Barbier reaction conditions [23], using a homoallylic alcohol as a reaction intermediate, followed by a Prins cyclization [2,24].

There are few examples in the literature that describe the formation of THP rings prepared via one-pot reactions. Among them, we cite the work of Yi and collaborators [25], who accidentally obtained a mixture of tetrahydropyran-4-ol and 4-bromotetrahydropyran compounds in the presence of indium metal.

On the other hand, in the search for sustainability and technologies that do not harm the environment, one of the routes being explored is the substitution of “traditional” organic solvents with ionic liquids. Due to strong interactions between the ions, they are not volatile and can exercise unexpected catalytic activity [26].

**Scheme 2:**
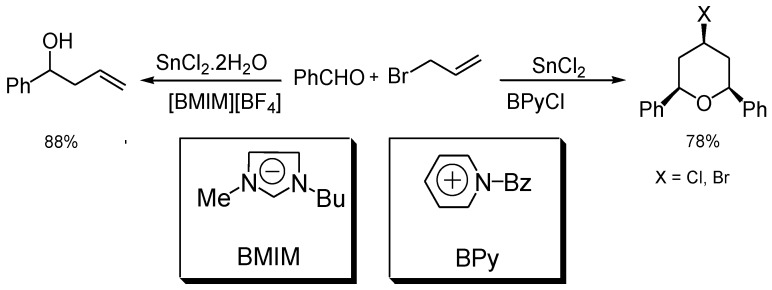
Different products obtained under Barbier reaction conditions in ionic liquids.

Recently, Zhao et al. [27] showed that ionic liquids containing halide anions promote the formation of THPs rings in good yields via a one-pot Barbier–Prins sequence of reactions; however, the reaction is sensitive to the presence of water in the tin (II) halide. In contrast, Tang et al. [28] subjected the Barbier reaction to using [BMIM][BF_4_] as the ionic liquid, with the homoallylic alcohol as the only product obtained with 88% yield for benzaldehyde (Scheme 2). Additionally, McCluskey [29] using [BMIM][PF_6_], performed the allylation of an aldehyde from tetraallylstannane and obtained only the homoallylic alcohol as the product in good yields. These observations drew our attention to further study the Barbier–Prins reaction under different conditions using [BMIM][PF_6_] as the solvent aiming at obtaining tetrahydropyrano rings.

In the present study, we propose the one-pot synthesis of 2,4,6-trisubstituted tetrahydropyran compounds promoted by ionic liquids [BMIM][PF_6_] and your role in the tandem Barbier–Prins reaction.

The ionic liquid [BMIM][PF_6_] was chosen because it has been routinely used based on the premise that the imidazolium does not have halide contamination as a result of its preparation [30]. In addition, it is a safe, environmentally friendly, immiscible with water, and especially for this reaction, there are no experimental discussions about its participation in Barbier–Prins reactions.

## 2. Results and Discussion

We started our studies with the reaction between benzaldehyde, allyl bromide, and stannous chloride dihydrate described in the Barbier protocol, using [BMIM][PF_6_] as a reaction medium under different conditions. The reaction formed products **4a** and **4b** by the Prins cyclization and the homoallylic alcohol **3** under certain conditions. The results are shown in Table 1.

We observed that an increased quantity of allyl bromide during the reaction led to an increased Prins product conversion (Entry 3). Interestingly, when the halogen atom of the allyl compound and the tin halide are different, two 2,4,6-trisubstituted tetrahydropyran compounds were formed, wherein THP chlorine is preferably formed in all tests performed in Table 1.

**Scheme 3:**
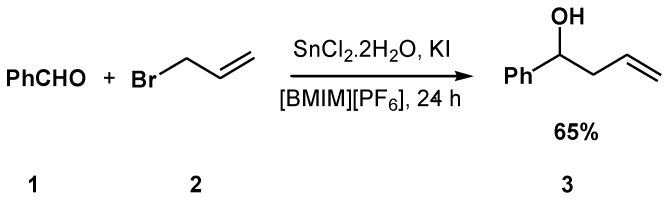
Reaction of **(1)** and **(2)** with KI in [BMIM][PF_6_].

The methodology developed by Houllemare [31], in which addition of potassium iodide (KI) accelerates the Barbier reaction to obtain homoallylic alcohols, was tested. Under these conditions, the presence of KI resulted in the lack of Prins product formation consequently only the homoallylic alcohol **3** was observed (Scheme 3).

We performed the same reaction with two other ionic liquids, using 1-Butyl-3-methylimidazolium (BMIM) as the cation and replaced the anion with [CF_3_CO_2_^−^] and [CF_3_SO_3_^−^]. The results of this study show the Barbier–Prins reaction in the five different ionic liquids with SnCl_2_.2H_2_O (Table 2).

As seen in Table 2, the tetrahydropyran compounds were obtained using the PF_6_ anion combined with the cation (BMIM) (Entry 3), presenting similar results to the work of Wang et al [27] (Table 2, Entry 2).

Li and coworkers [28] obtained the homoallylic alcohol **(3)** using the liquid [BMIM][BF_4_] (Table 2, entry 1). We also performed this same reaction under the conditions of entry 3 (Table 2). The results showed similar behavior to that found by the authors with only the homoallylic alcohol obtained.

Of the ionic liquids applied in Table 2, [BMIM][PF_6_] is the only one that is immiscible with water besides the only one provides formation of the THP product mixture, even when using the hydrated salt (SnCl_2_.2H_2_O). Apart from our results, Wang and coworkers [27], when using the ionic liquid BPyCl under the same conditions, only the homoallylic alcohol was obtained in a 93% yield on the other hand, THPs were only observed when using anhydrous SnCl_2_. In view of these results, we indicate that the use of an immiscible ionic liquids with water influenced the products formed from the Barbier–Prins reaction.

The ionic liquids from [CF_3_CO_2_^-^] and [CF_3_SO_3_^-^], as well as the [BF_4_^−^] anions (Entry 1, 4, and 5), did not generate the Prins product, as observed for the PF_6_ anion. The ionic liquids [BMIM][CF_3_CO_2_] and [BMIM][CF_3_SO_3_] behaved similar to [BMIM][BF_4_] [28] and led to the exclusive formation of homoallylic alcohol with yields of 48% and 82%, respectively.

We investigated the Barbier–Prins reaction in [BMIM][PF_6_] using other metallic sources, such as Zn and Sn, which are also used in the Barbier reaction [23] (Table 3).

The reactions with metallic Zn and metallic Sn did not lead to the formation of any of the products **3**, **4a** and **4b** (entries 2 and 3). Using SnCl_4_ and AlCl_3_ as a Lewis acid, when introduced to the reaction to activate the carbonyl group and accelerate the reaction, did not influence the conversion or the reaction time (entries 4 and 5). Substituting SnCl_2_.2H_2_O for SnBr_2_ led to the exclusive formation of the Prins product **4b** with excellent conversion (entry 6).

In the next phase of this study, we applied reaction conditions better established for other aldehydes. The reaction was studied with SnCl_2_·2H_2_O and SnBr_2_, and the results are shown in Table 4.

In general, reactions with aromatic aldehydes with electron withdrawing groups (entries 3–6) and electron donating group (entries 7, 8) at the 4-position presented good yields, similar to the reaction with the aromatic aldehyde without any substitution. However, the reaction with 4-NO_2_-benzaldehyde (entries 9, 10), under the performed conditions, does not lead to formation of the homoallylic alcohol or Prins product, even after 48 h of reaction time. These reactions were performed with a slight warming of 40 degrees, obtaining the Prins product in a 40% yield, both when it was used with SnCl_2_ or SnBr_2_. The results from the work of Slaton [32] show that the performance of the homoallylic alcohol formed from the 4-NO_2_-benzaldehyde is lower compared with other substituents. Therefore, the reaction yield is decreased, possibly because the homoallylic alcohol formed in the reaction is in a small quantity. The reaction of the aliphatic aldehyde (entries 11, 12) has a similar behavior to aromatic aldehydes (entries 1–8).

For all 2,4,6-trisubstituted tetrahydropyran compounds, the cis compound was obtained. The structures of the compounds were confirmed by comparison with the literature data, where the tetrahydropyrans obtained followed the same standards for the chemical shift data and the NMR coupling constants [33].

**Figure 1:**
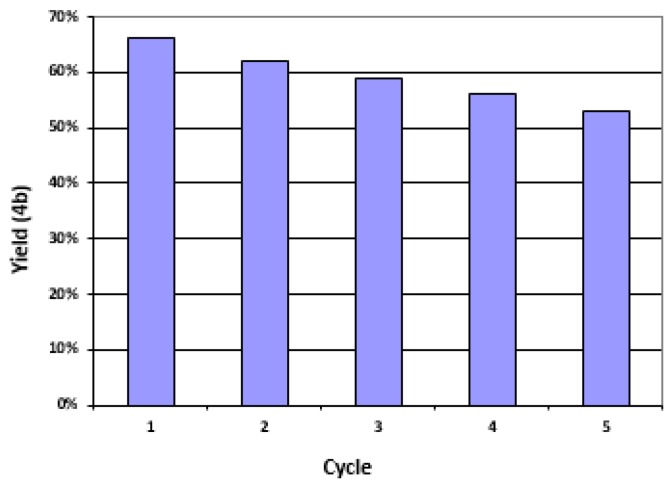
Reuse of ionic liquid [BMIM][PF_6_] in the synthesis of **4b** at room temperature.

In regard to [BMIM][PF_6_] being completely immiscible with water, it is easy to separate it from the reaction medium and recycle it. The Figure 1 shows the yields of the one-pot Barbier–Prins cyclization combined reaction, using benzaldehyde under the conditions of entry 6 in Table 3.

As shown in Figure 1, the ionic liquid [BMIM][PF_6_] was reused consecutively, up to five times, with little decrease in tetrahydropyran yield, ranging from 66% to 53%.

Later, we replaced the ionic liquids with water, using the salt KPF_6_ to test the influence of PF_6_^-^ in the reaction. It was found that the product conversion ratio is increased when the amount of water decreased (Table 5, entries 2 and 3). However, upon removing the solvent (water), the reaction did not proceed, suggesting that the water is part of the reaction (Table 5, entry 1). Water should provide the energy required to cause the dissociation of KPF_6_. Without water, the dissociation of PF_6_^-^ cannot occur.

We repeated the reaction using KPF_6_ with other solvents (Table 5, entry 4–7) for example when toluene was used (entry 4) no product is formed. However the use of polar solvents as ethanol (entry 5), acetonitrile (entry 6) and dichloromethane (entry 7) enabled yields were very similar at approximately 30%.

From the experiments in Table 5, we verify that the KPF_6_ salt is an important reactant in the Barbier–Prins reaction in aqueous medium, with the best result obtained when only 10 µL of water was used as the reaction solvent (Entry 3). However, it would still be necessary to investigate this working step, in which the KPF_6_ salt was acting as a catalyst in the Barbier reaction or the Prins reaction. For this, we conducted another experiment (Table 6) to investigate the performance of KPF_6_ and SnBr_2_ for the Prins cyclization reaction between the homoallylic alcohol and benzaldehyde.

**Scheme 4:**
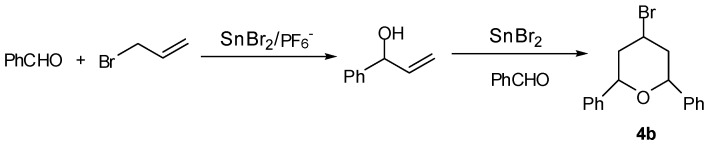
The role of ionic liquid [BMIM][PF_6_] and SnBr_2_ in the THP-**4b** synthesis.

The product 4b was obtained in good yield in only an 8 h reaction by Prins cyclization from allylic alcohol (Table 6, entries 1 and 2) moreover it can be observed that without KPF_6_ the same reaction showed similar yields. In fact, we concluded with the Barbier reaction is very slow without KPF_6_, therefore this salt acts as a catalyst in the Barbier reaction and has no influence on the Prins cyclization. On the other hand, SnBr_2_ in slight excess acts as Lewis acid in the Prins cyclization (Scheme 4).

## 3. Experimental

### 3.1. Synthesis of 2,4,6-Trisubstituted Tetrahydropyran Compounds (General Procedure)

A mixture of aldehyde (0.33 mmol), allyl bromide (1.32 mmol), SnBr_2·_2H_2_O (0.5 mmol) and [BMIM][PF_6_] (0.5 mL) was agitated at room temperature for 8 h. The reaction mixture was extracted in diethyl ether (three times) and then an aqueous solution of HCl was added in the combined ether phases. The organic phase was separated and dried with anhydrous Na_2_SO_4_.– The solvent was removed under vacuum, and the THPs product was purified by silica gel column chromatography, using a mixture of ethyl acetate and petroleum ether (1:50) as an eluent; a colorless solid was obtained. An aliquot the crude product was analyzed with gas chromatography for obtained the conversion of reaction and ratio between chlorinated and brominated compounds. The NMR spectra (^1^H and ^13^C) for compounds **4b**–**9b** is shown in the Supplementary Materials.

### 3.2. Characterization Data *[32a]*

*4-Bromo-tetrahydro-2,6-diphenyl-2H-pyran* (**4b**): ^1^H NMR (200 MHz, CDCl_3_) δ = 7.35 (m, 10H, ArH), 4.57 (d, *J* = 12 Hz, 2H, H_2ax_ e H_6ax_), 4.44 (m, 1H, H_4ax_), 2.56 (m, 2H, H_3ax_ e H_5ax_), 2.11 (m, 2H, H_3eq_ e H_5eq_). ^13^C NMR (50 MHz, CDCl_3_) δ = 145.13, 132.34, 131.68, 129.70, 83.66, 50,08, 49.03. Yield 73%.

*4-Bromo-2,6-bis(4-fluorophenyl)-tetrahydro-2H-pyran* (**5b**): ^1^H NMR (500 MHz, CDCl_3_) δ = 7.38 (m, 4H, ArH), 7.06 (m, 4H, ArH), 4.55 (m, *J* = 10.0 Hz, 2H, H_2ax_ e H_6ax_), 4.41 (m, 1H, H_4ax_), 2.55 (m, 2H, H_3ax_ e H_5ax_), 2.09 (m, 2H, H_3eq_ e H_5eq_); ^13^C NMR (125 MHz, CDCl_3_) δ = 164.69, 159.90, 136.85, 136.78, 127.55, 127.39, 115.50, 115.08, 79,08, 45,48, 44.92. Yield 71%.

*4-Bromo-2,6-bis(4-chlorophenyl)-tetrahydro-2H-pyran* (**6b**): ^1^H NMR (500 MHz, CDCl_3_) δ = 7.34 (m, 8H, ArH), 4.54 (dd, *J* = 10.0, 2H, 8.0, H_2ax_ e H_6ax_), 4.42 (m, 1H, H_4ax_), 2.54 (m, 1H, H_3ax_ e H_5ax_), 2.06 (m, 1H, H_3eq_ e H_5eq_); ^13^C NMR (125 MHz, CDCl_3_) δ = 139.47, 133.59, 128.66, 127.14, 79.06, 45.27, 44.81. Yield 73%.

*4-Bromo-tetrahydro-2,6-dip-tolyl-2H-pyran* (**7b**): ^1^H NMR (500 MHz, CDCl_3_) δ = 7.31 (m, 8H, ArH), 4.54 (dd, *J* = 10.0 Hz, 2H, H_2ax_ e H_6ax_), 4.45 (m, 1H, H_4ax_), 2.55 (m, 1H, H_3ax_ e H_5ax_), 2,36 (s, 6H), 2.12 (m, 3H, H_3eq_ e H_5eq_); ^13^C NMR (125 MHz, CDCl_3_) δ = 133.48, 132.51, 124.16, 120.99, 74.73, 41.55, 40.23, 16,25. Yield 60%.

*4-Bromo-tetrahydro-2,6-bis(4-nitrophenyl)-2H-pyran* (**8b**): ^1^H NMR (500 MHz, CDCl_3_) δ = 8.28 (m, 4H, ArH), 7.63 (m, 4H, ArH), 4.75 (dd, *J* = 10.0, 2H, H_2ax_ e H_6ax_), 4.49 (m, 1H, H_4ax_), 2.66 (m, 2H, H_3ax_ e H_5ax_), 2.12 (m, 2H, H_3eq_ e H_5eq_); ^13^C NMR (125 MHz, CDCl_3_) δ = 147.55, 126.39, 123.84, 78.69, 44.32, 43.87. Yield 40%.

*4-Bromo-tetrahidro-2,6-dihexyltetrahidropyrano* (**9b**): ^1^H NMR (500 MHz, CDCl_3_): δ = 4.12 (m, H_4ax_), 3.21 (m, H_2ax_ e H_6ax_), 2.17 (dd, *J* = 10.0 Hz, H_3eq_ e H_5eq_), 1.61 (m, H_3ax_ e H_5ax_), 1.24 (m, 24H, (CH_2_)_6_), 0.83 (t, 6H, CH_3_). ^13^C NMR (125 MHz, CDCl_3_): δ = 77.52, 47.33, 43.43, 35.74, 31.65, 29.03, 25.34, 22.45, 13.94. Yield 60%.

## 4. Conclusions

In this study, we showed tandem reactions combining Barbier and Prins for produces stetrahydropyrans compounds from an allylbromide, tin halide, and aldehyde, promoted by the ionic liquid [BMIM][PF_6_] with moderate to good yields in just an 8 h reaction. With other parallel experiments, we can see how the reagents work in two reactions. In addition, the PF_6_^-^ anion presents in ionic liquid besides contributing the ionic medium to the reaction mechanism acts to accelerate the reaction and only ionic medium influence on the Prins cyclization. Slight excess of SnBr_2_ under the reaction conditions of the Barbier reaction leads to the formation of the product of THPs, thus acting as a catalyst for Prins cyclization.

## Figures and Tables

**Table 1:**
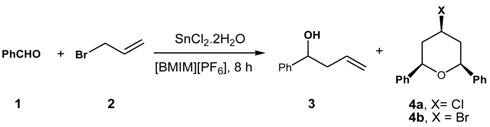
Reaction between benzaldehyde (1) and allyl bromide **(2)** promoted by SnCl_2_·2H_2_O in [BMIM][PF_6_].

Entry ^a^	1:2 Ratio	Conversion (%) ^b^	Ratio ^b^ (3:4a:4b)%
1	1:1.5	12	0:92:8
2	1:3	51	0:84:16
3	1:4	92	0:73:27
4	1:6	92	0:69:31

^a^ Allyl bromide (1.32 mmol), Benzaldehyde (0.33 mmol), SnCl_2_·2H_2_O (0.5 mmol), [BMIM][PF_6_] (2.4 mmol), Reaction time: 8 h, room temperature. ^b^ Determined by GC/FID.

**Table 2:**
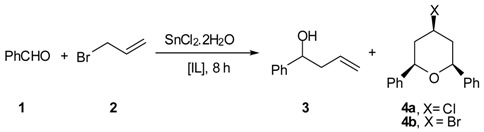
Reaction of benzaldehyde **(1)** with allyl bromide **(2)** in different ionic liquids.

Entry	Ionic Liquid	Isolated Yield (%)	Ratio (3:4a:4b) %
1 ^a^	[BMIM][BF_4_]	88	100:0:0
2	[BPy][Br]^27^	77	0:76:24
3 ^a^	[BMIM][PF_6_]	75	0:73:27
4 ^a^	[BMIM][CF_3_CO_2_]	48	100:0:0
5 ^a^	[BMIM][CF_3_SO_3_]	85	100:0:0

^a^ Allyl bromide (1,32 mmol), Benzaldehyde (0,33 mmol), SnCl_2_.2H_2_O (0.5 mmol), IL (2.4 mmol), room temperature, 8 h.

**Table 3:**
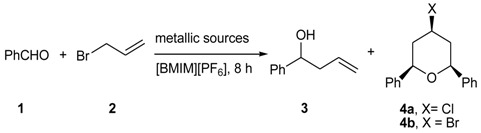
Reaction of benzaldehyde **(1)** with allyl bromide **(2)** in [BMIM][PF_6_], varying the metallic sources.

Entry ^a^	Metallic Sources	Conversion (%) ^c^	Ratio 3:4a:4b
1	SnCl_2_·2H_2_O	92	0:73:27
2	Zn	0	0:0:0
3	Sn	0	0:0:0
4	SnCl_2_·2H_2_O/SnCl_4_	85	0:82:18
5 ^b^	SnCl_2_·2H_2_O/AlCl_3_	86	0:83:17
6	SnBr_2_	90	0:0:100

Allyl bromide (1.32 mmol), Benzaldehyde (0.33 mmol), [BMIM][PF_6_] (2.4 mmol), ambient temperature, 8 h. ^a^ Metallic sources (0.5 mmol), ^b^ SnCl_4_ (0.16 mmol) or AlCl_3_ (0.16 mmol), ^c^ Determined by GC/FID.

**Table 4:**
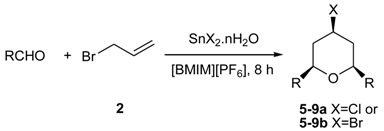
Synthesis of several Tetrahydropyrans (THPs) varying tin halides promoted by [BMIM][PF_6_].

Entry	Aldehyde	Tin Halide	Yield Isolated (%)	Ratio
1 ^a^	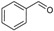	SnCl_2_.2H_2_O	75	**4a**:**4b** (73:27)
2 ^a^	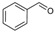	SnBr_2_	73	**4b**
3 ^a^	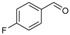	SnCl_2_.2H_2_O	71	**5a**:**5b** (63:37)
4 ^a^	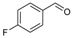	SnBr_2_	70	**5b**
5 ^a^	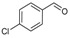	SnCl_2_.2H_2_O	71	**6a**:**6b** (62:38)
6 ^a^	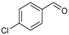	SnBr_2_	72	**6b**
7 ^a^	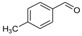	SnCl_2_.2H_2_O	60	**7a**:**7b** (65:35)
8 ^a^	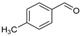	SnBr_2_	62	**7b**
9 ^b^	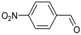	SnCl_2_.2H_2_O	40	**8a**:**8b** (65:35)
10 ^b^	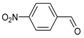	SnBr_2_	45	**8b**
11 ^a^	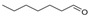	SnCl_2_.2H_2_O	61	**9a**:**9b**(60:40)
12 ^a^	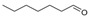	SnBr_2_	60	**9b**

Allyl bromide (1.32 mmol), Benzaldehyde (0.33 mmol), SnCl_2_·2H_2_O (0.5 mmol), [BMIM][PF_6_] (2.4 mmol), 8 h, ^a^ room temperature, ^b^ Temperature 40 °C.

**Table 5:**
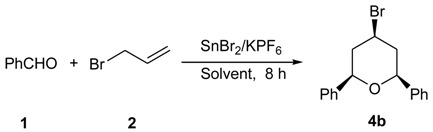
Reaction of benzaldehyde **(1)** with allyl bromide **(2)** promoted by KPF_6._

Entry	Solvent	Yield Isolated (%)
1	-	0
2	H_2_O/0.5 mL	43
3	H_2_O/10 µL	63
4	Toluene/0.5 mL	0
5	Ethanol/0.5 mL	31
6	Acetonitrile/0.5 mL	34
7	CH_2_Cl_2_/0.5 mL	37

Conditions: Allyl bromide (1.32 mmol), Benzaldehyde (0.33 mmol), SnBr_2_. (0.5 mmol), 24 h, room temperature.

**Table 6:**
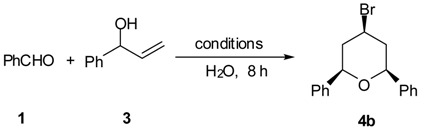
Reaction of benzaldehyde **(1)** with homoallylic alcohol **(3)** promoted by SnBr_2_.

Entry	Condition	Yield Isolated (%)
1	SnBr_2_2H_2_O/KPF_6_	78
2	SnBr_2_.2H_2_O	77

Conditions: 1-phenylbut-3-en-1-ol (0.33 mmol), Benzaldehyde (0.33 mmol), SnBr_2_ (0.5 mmol), KPF_6_ (0.16 mmol), H_2_O (10 µL), room temperature, 8 h.

## Supplementary Materials

The following are available online at https://www.mdpi.com/1420-3049/24/11/2084/s1.
