# Supplementary material for: The Role Ionic Liquid [BMIM][PF6] in One-Pot Synthesis of Tetrahydropyran Rings through Tandem Barbier–Prins Reaction
Source: Molecules. 2019 May 31;24(11):2084. doi: 10.3390/molecules24112084 (PMC6600659; doi:10.3390/molecules24112084)
Supplement: Supplementary file 1 [file molecules-24-02084-s001.pdf]

The Role Ionic Liquid [BMIM][PF_6] in One-Pot Synthesis of Tetrahydropyran Rings through Tandem Barbier–Prins Reaction

Poliane K. Batista, João Marcos G. de O. Ferreira, Fabio P. L. Silva, Mario L. A. A Vasconcellos and Juliana A. Vale *

Departamento de Química, Universidade Federal da Paraíba, Cidade Universitária, 58051-900, João Pessoa-PB, Brazil; polianekarenine@gmail.com (P.K.B.); profjoaomarcos@hotmail.com (J.M.G.d.O.F.); pedrosalinssilva@gmail.com (F.P.L.S.); mlaav00@gmail.com (M.L.A.A.V.)

* Correspondence: julianadqf@yahoo.com.br; Tel.:+558332167433

[a]*Departamento de Química, Universidade Federal da Paraíba, Cidade Universitária, 58051-900, João Pessoa-PB*

4-bromo-tetrahydro-2,6-diphenyl-2H-pyran (4b): ^{1}H NMR (200 MHz, $CDCl_3$) δ = 7.35 (m, 10H, 10H aromatic), 4.57 (d, J = 12 Hz, 2H, H_2 ax e H_6 ax), 4.44 (m, 1H, H_4 ax), 2.56 (m, 2H, H_3 ax e H_5 ax), 2.13 (m, 2H, H_3 eq e H_5 eq). ^{13}C NMR (50 MHz, $CDCl_3$) δ = 145.13, 132.63, 131.68, 129.70, 83.66, 50,08, 49.03.

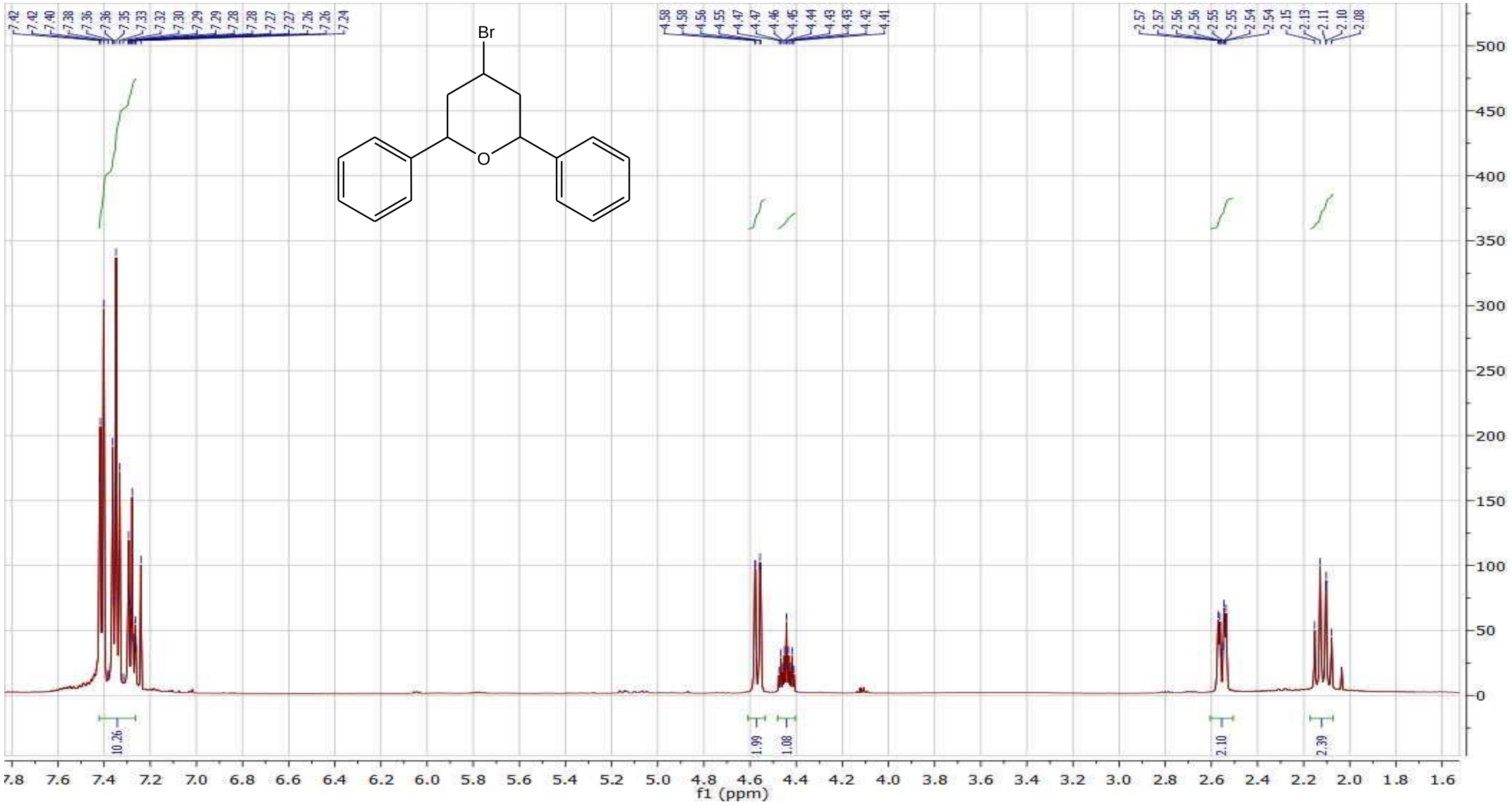

Espectro S1 - Espectro de RMN ^{1}H ($CDCl_3$, 200 MHz) do 4-bromo-tetrahydro-2,6-diphenyl-2H-pyran (4b)

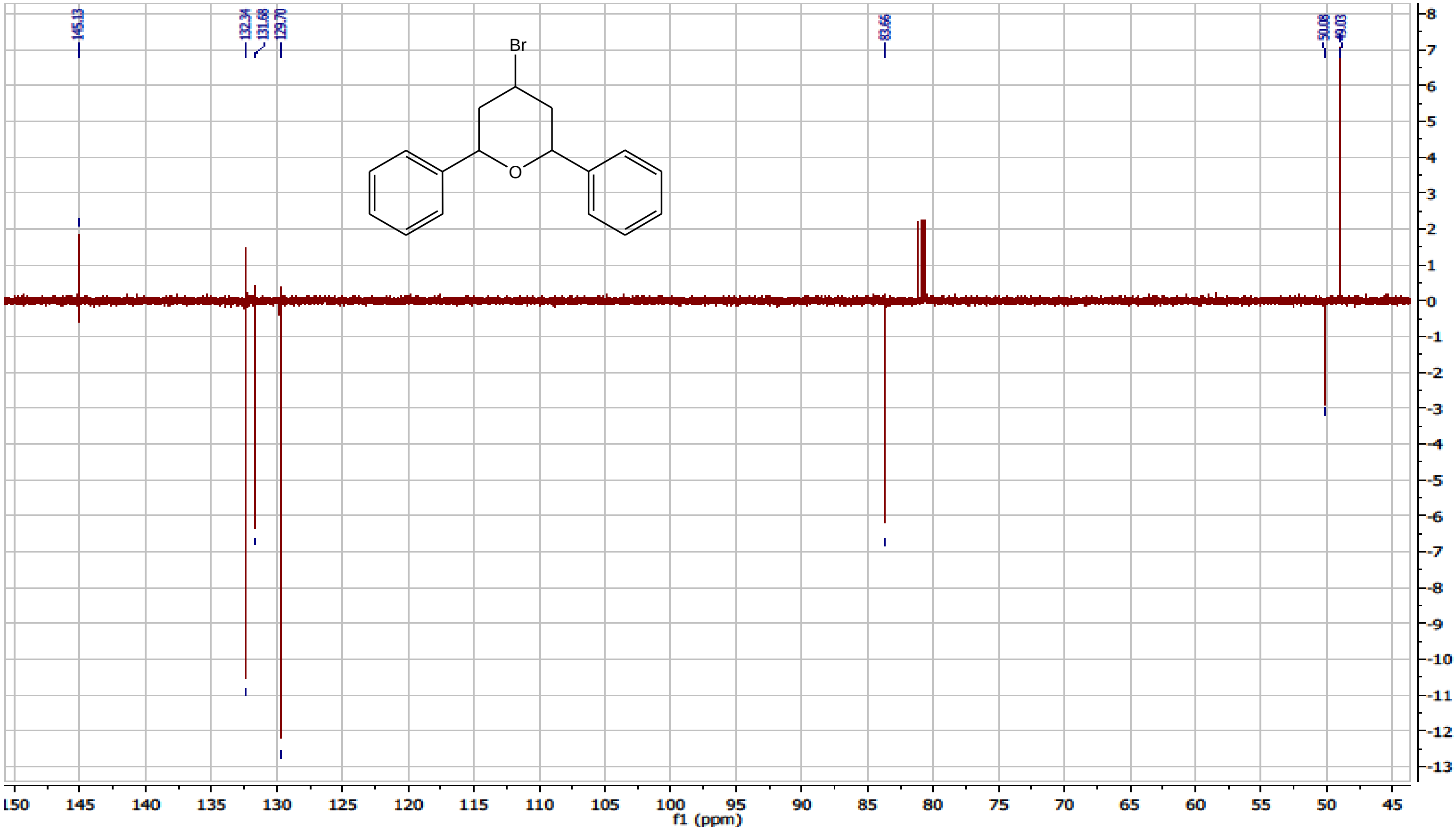

Espectro S2 - Espectro de ^{13}C RMN ($CDCl_3$, 50 MHz) do 4-bromo-tetrahydro-2,6-diphenyl-2H-pyran (4b)

4-bromo-2,6-bis(4-fluorophenyl)-tetrahydro-2H-pyran (5b): ^{1}H NMR (500 MHz, $CDCl_3$) δ = 7.38 (m, 4H, 4H aromatic), 7.06 (m, 4H, 4H aromatic), 4.55 (m, J 10.0, 2H, H_2 ax e H_6 ax), 4.41 (m, 1H, H_4 ax), 2.55 (m, 2H, H_3 ax e H_5 ax), 2.09 (m, 2H, H_3 eq e H_5 eq); ^{13}C NMR (125 MHz, $CDCl_3$) δ = 158.55, 156.59, 122.79, 122.72, 110.68, 110.51, 74,44, 40,76, 40.25.

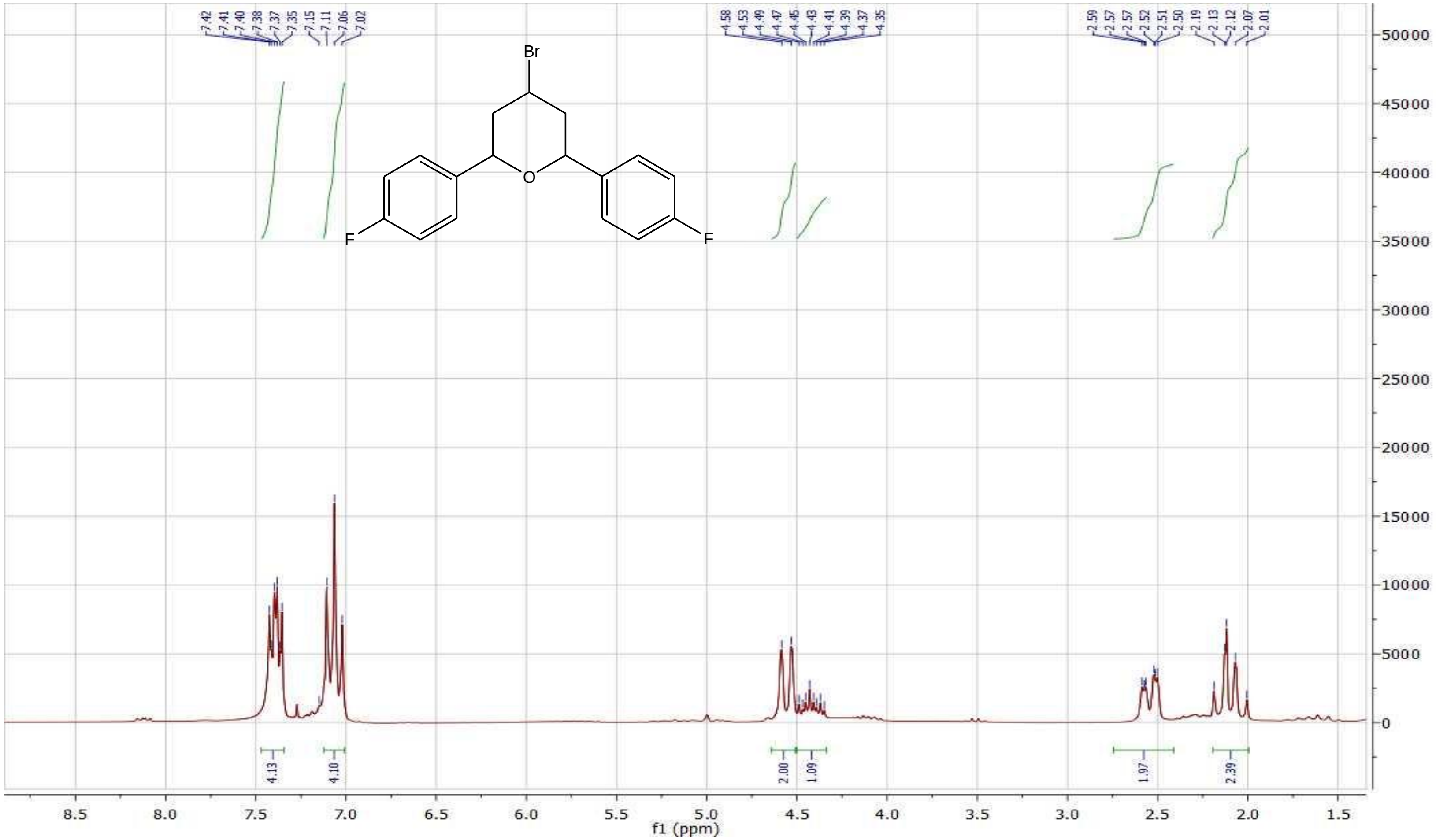

Espectro S3 - Espectro de RMN ^{1}H ($CDCl_3$, 500 MHz) do 4-bromo-2,6-bis(4-fluorophenyl)-tetrahydro-2H-pyran (5b)

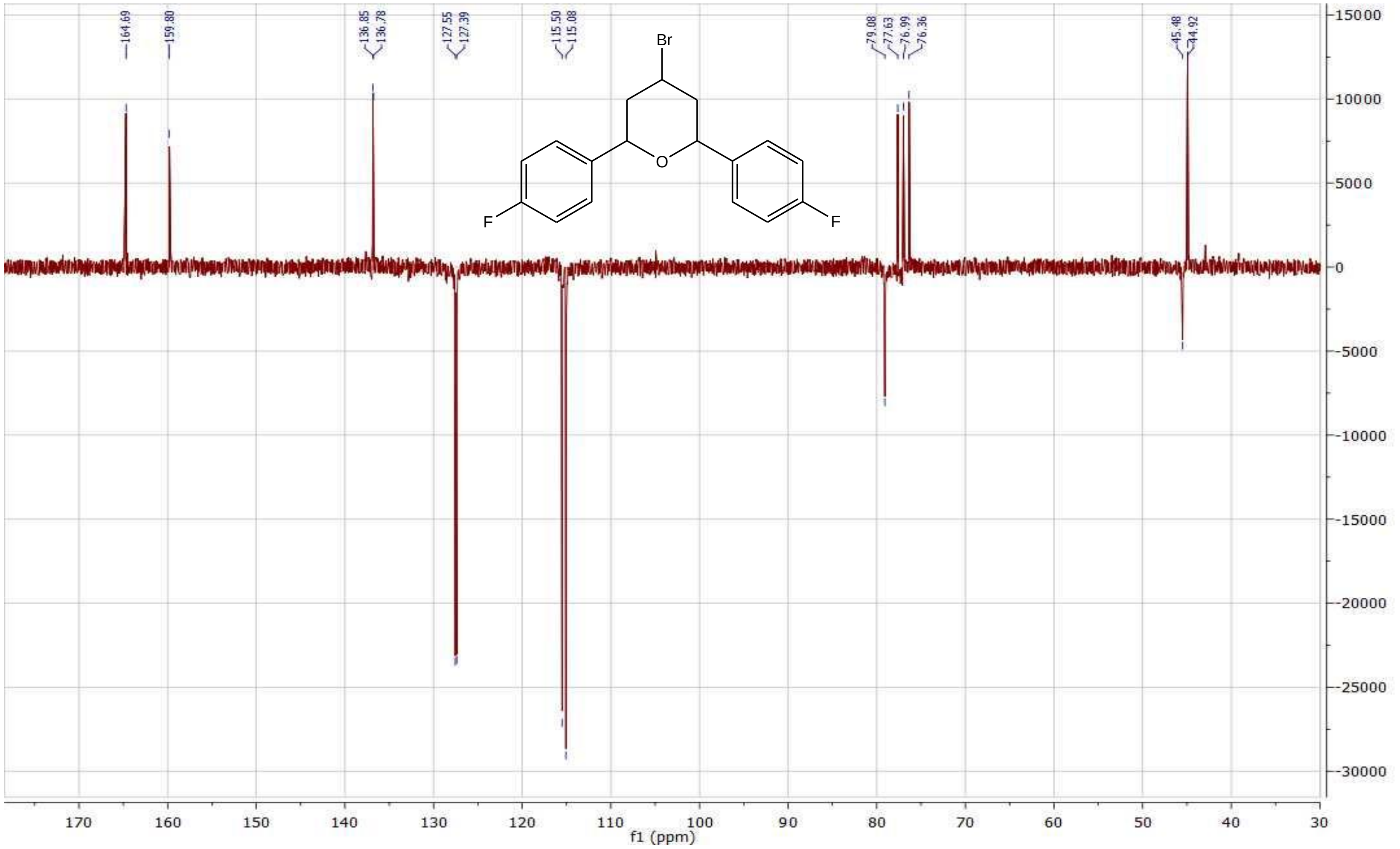

Espectro S4 - Espectro de RMN ^{13}C ($CDCl_3$, 125 MHz) do 4-bromo-2,6-bis(4-fluorophenyl)-tetrahydro-2H-pyran (5b)

4-bromo-2,6-bis(4-chlorophenyl)-tetrahydro-2H-pyran (6b): ^{1}H NMR (500 MHz, $CDCl_3$) δ = 7.34 (m, 8H, 8H aromatic), 4.54 (dd, J = 10.0, 2H, 8.0, H_2 ax e H_6 ax), 4.42 (m, 1H, H_4 ax), 2.54 (m, 1H, H_3 ax e H_5 ax), 2.06 (m, 1H, H_3 eq e H_5 eq); ^{13}C NMR (125 MHz, $CDCl_3$) δ = 139.47, 133.59, 128.66, 127.14, 79.06, 45.27, 44.81.

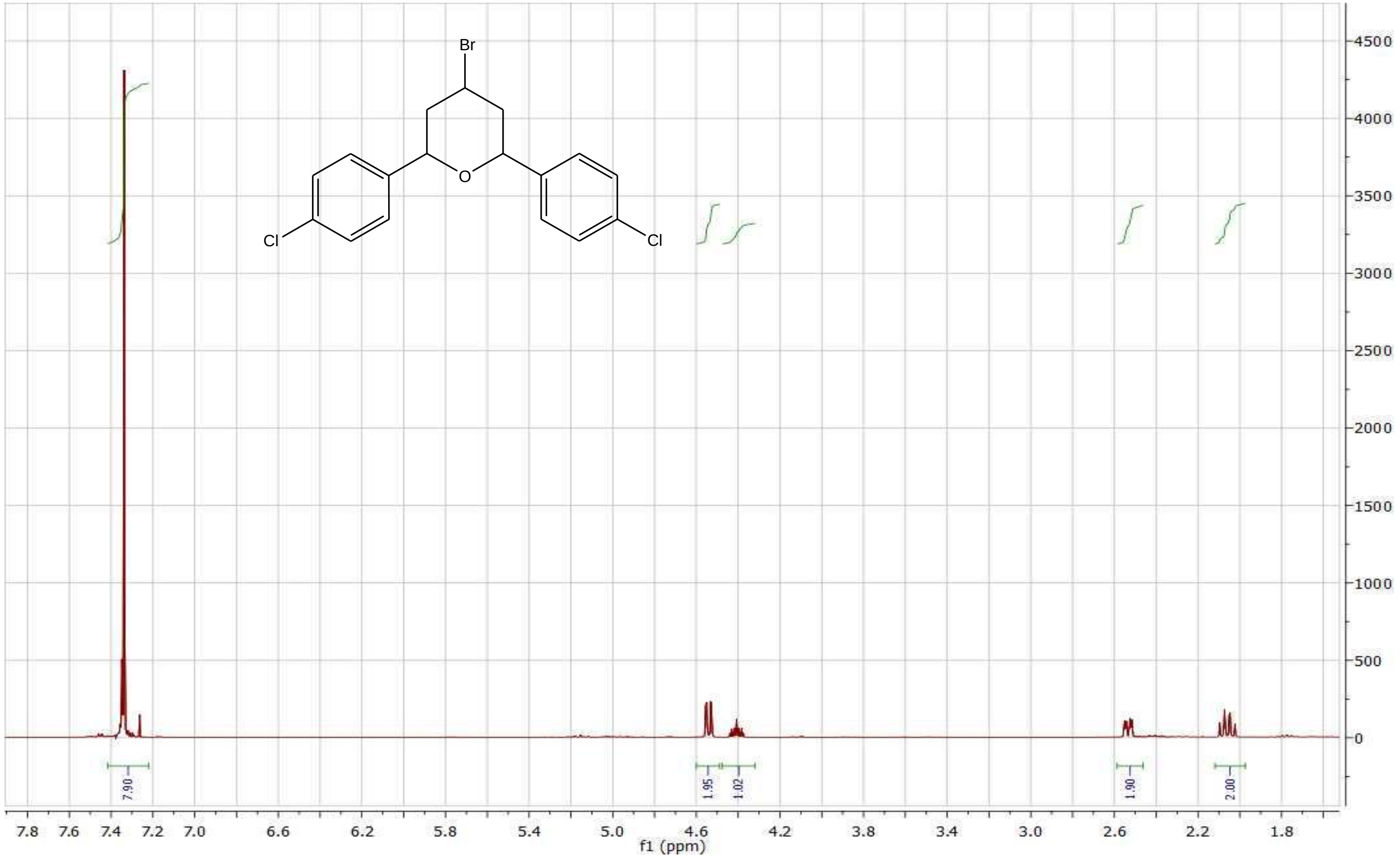

Espectro S5 - Espectro de RMN ^{1}H ($CDCl_3$, 500 MHz) do 4-bromo-2,6-bis(4-chlorophenyl)-tetrahydro-2H-pyran) (6b)

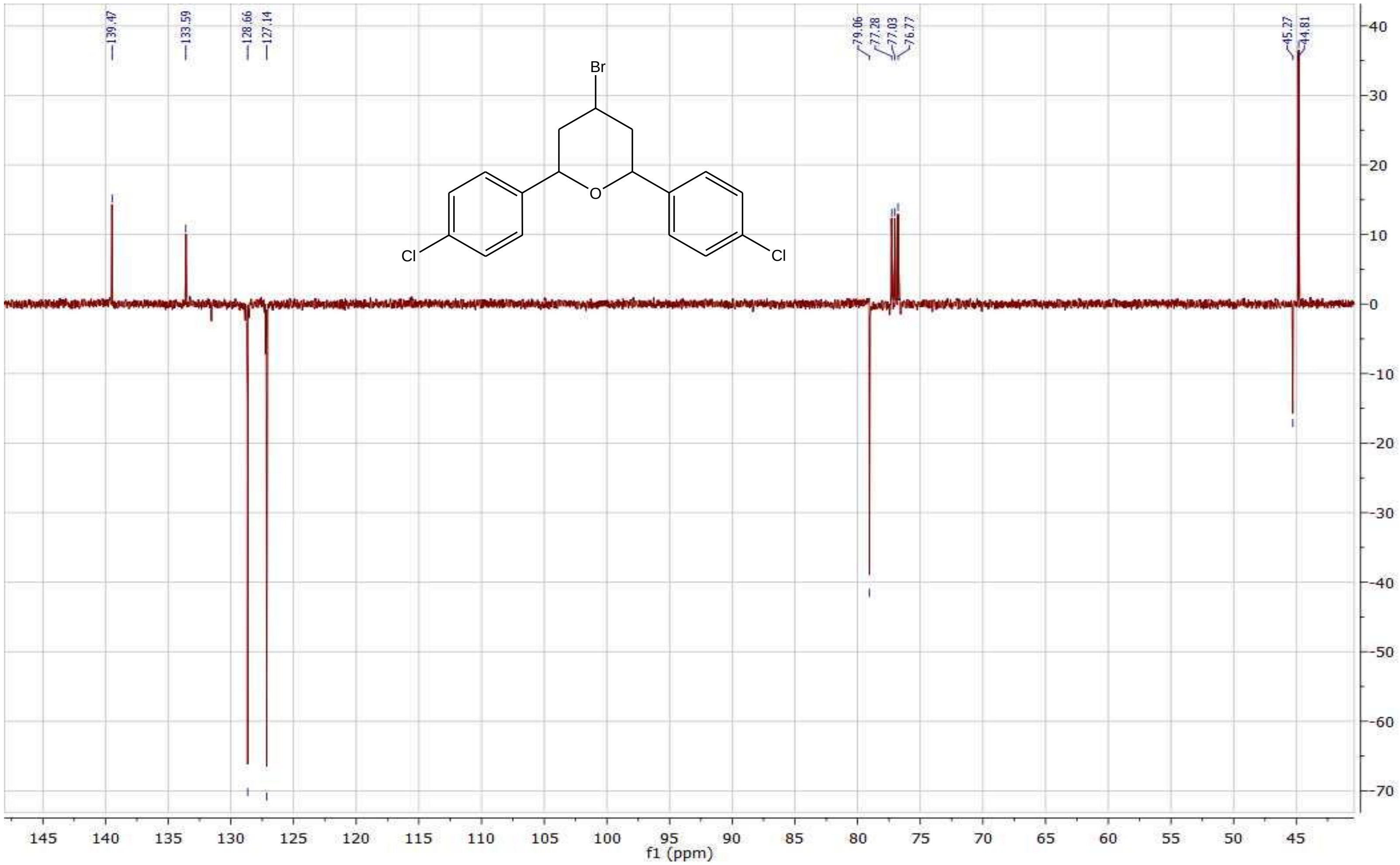

EspectroS6 - Espectro de RMN ^{13}C ($CDCl_3$, 125 MHz) do 4-bromo-2,6-bis(4-chlorophenyl)-tetrahydro-2H-pyran) (6b)

4-bromo-tetrahydro-2,6-dip-tolyl-2H-pyran (7b): ^{1}H NMR (500 MHz, $CDCl_3$) δ = 7.31 (m, 8H, 8H aromatic), 4.54 (dd, J = 10.0 Hz, 2H, H_2 ax e H_6 ax), 4.45 (m, 1H, H_4 ax), 2.55 (m, 1H, H_3 ax e H_5 ax), 2,36 (s, 6H), 2.12 (m, 3H, H_3 eq e H_5 eq); ^{13}C NMR (125 MHz, $CDCl_3$) δ = 133.48, 132.51, 124.16, 120.99, 74.73, 41.55, 40.23,16,25.

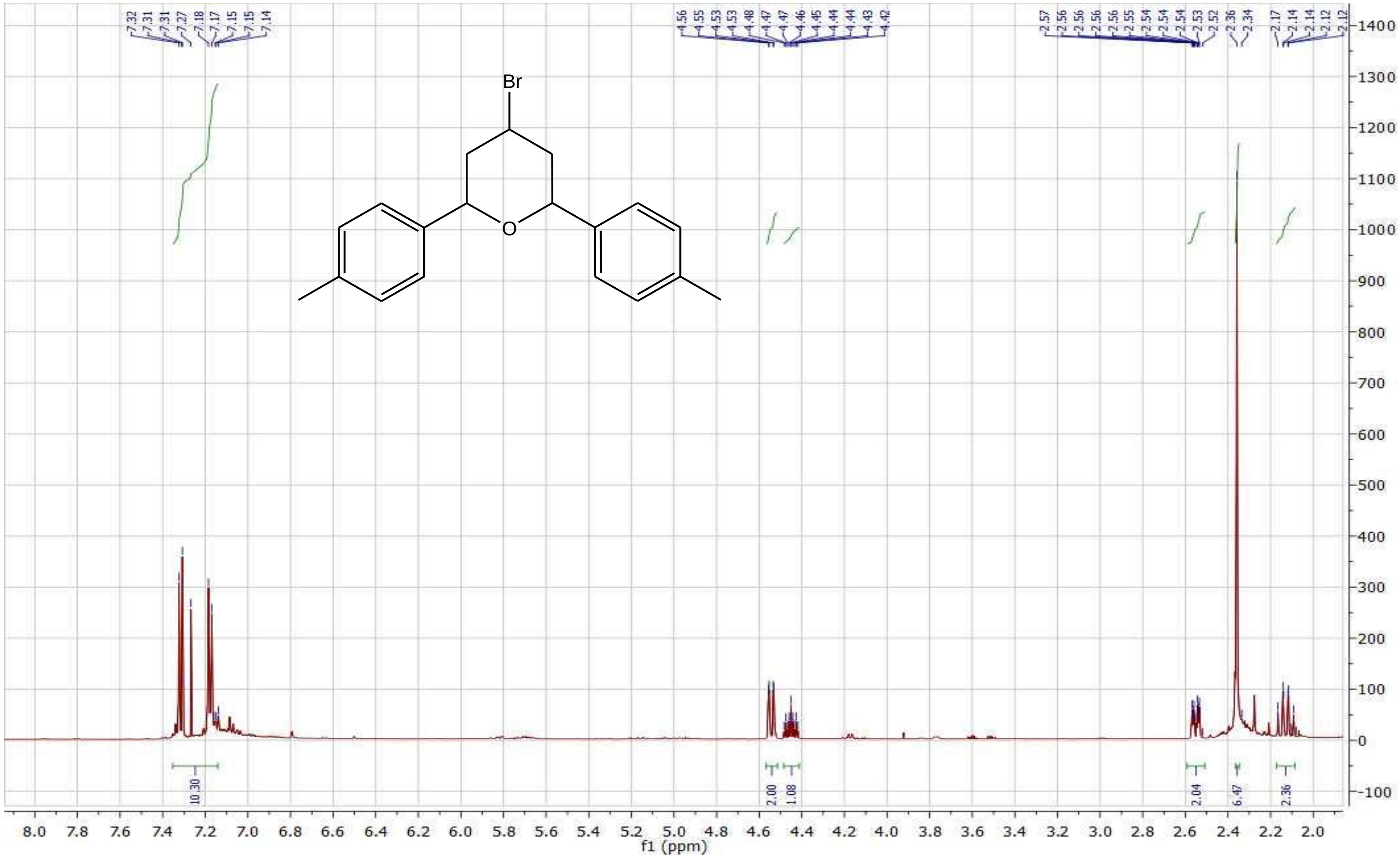

Espectro S7 - Espectro de RMN ^{1}H ($CDCl_3$, 500 MHz) do 4-bromo-tetrahydro-2,6-dip-tolyl-2H-pyran (7b)

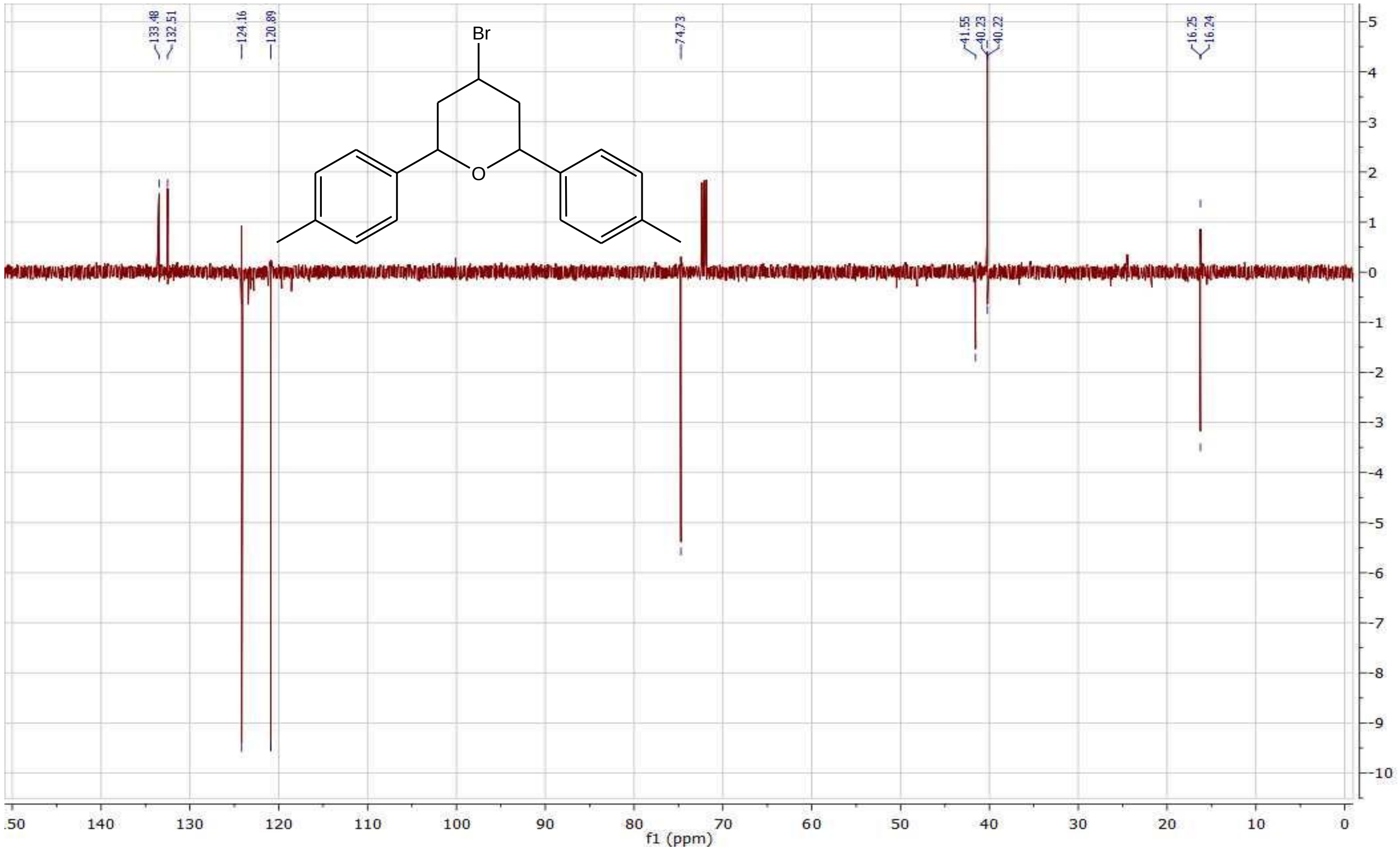

Espectro S8 - Espectro de RMN ^{13}C ($CDCl_3$, 125 MHz) do 4-bromo-tetrahydro-2,6-dip-tolyl-2H-pyran (7b)

4-bromo-tetrahydro-2,6-bis(4-nitrophenyl)-2H-pyran (8b): ^{1}H NMR (500 MHz, $CDCl_3$) δ = 8.28 (m, 4H, 4H aromatic), 7.63 (m, 4H, 4H aromatic), 4.75 (dd, J = 10.0, 2H, H_2 ax e H_6 ax), 4.49 (m, 1H, H_4 ax), 2.66 (m, 2H, H_3 ax e H_5 ax), 2.12 (m, 2H, H_3 eq e H_5 eq); ^{13}C NMR (125 MHz, $CDCl_3$) δ = 147.55, 126.44, 126.44, 123.87, 78.72, 44.36, 43.96.

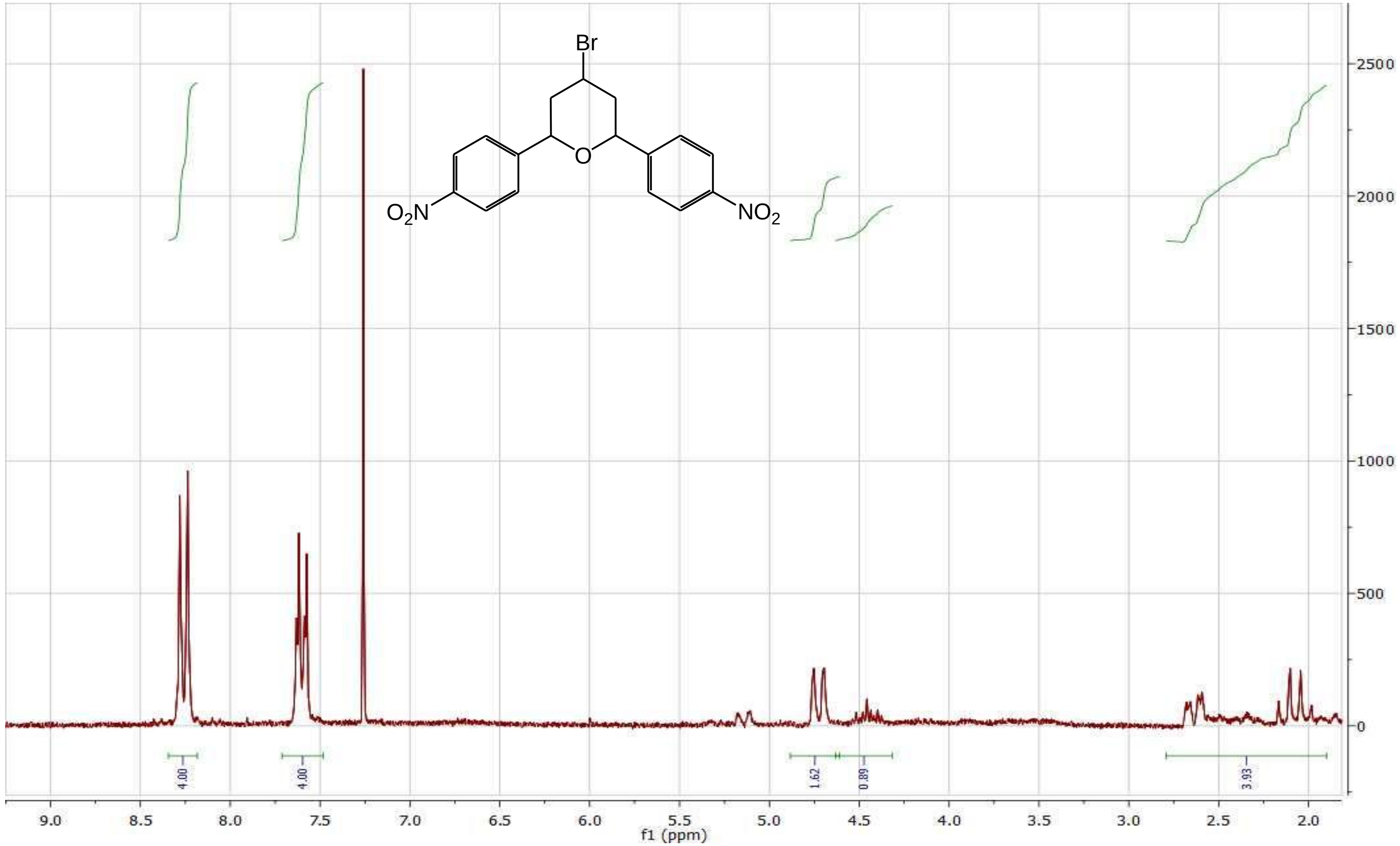

Espectro S9 - Espectro de RMN ^{1}H ($CDCl_3$, 500 MHz) do 4-bromo-tetrahydro-2,6-bis(4-nitrophenyl)-2H-pyran (8b)

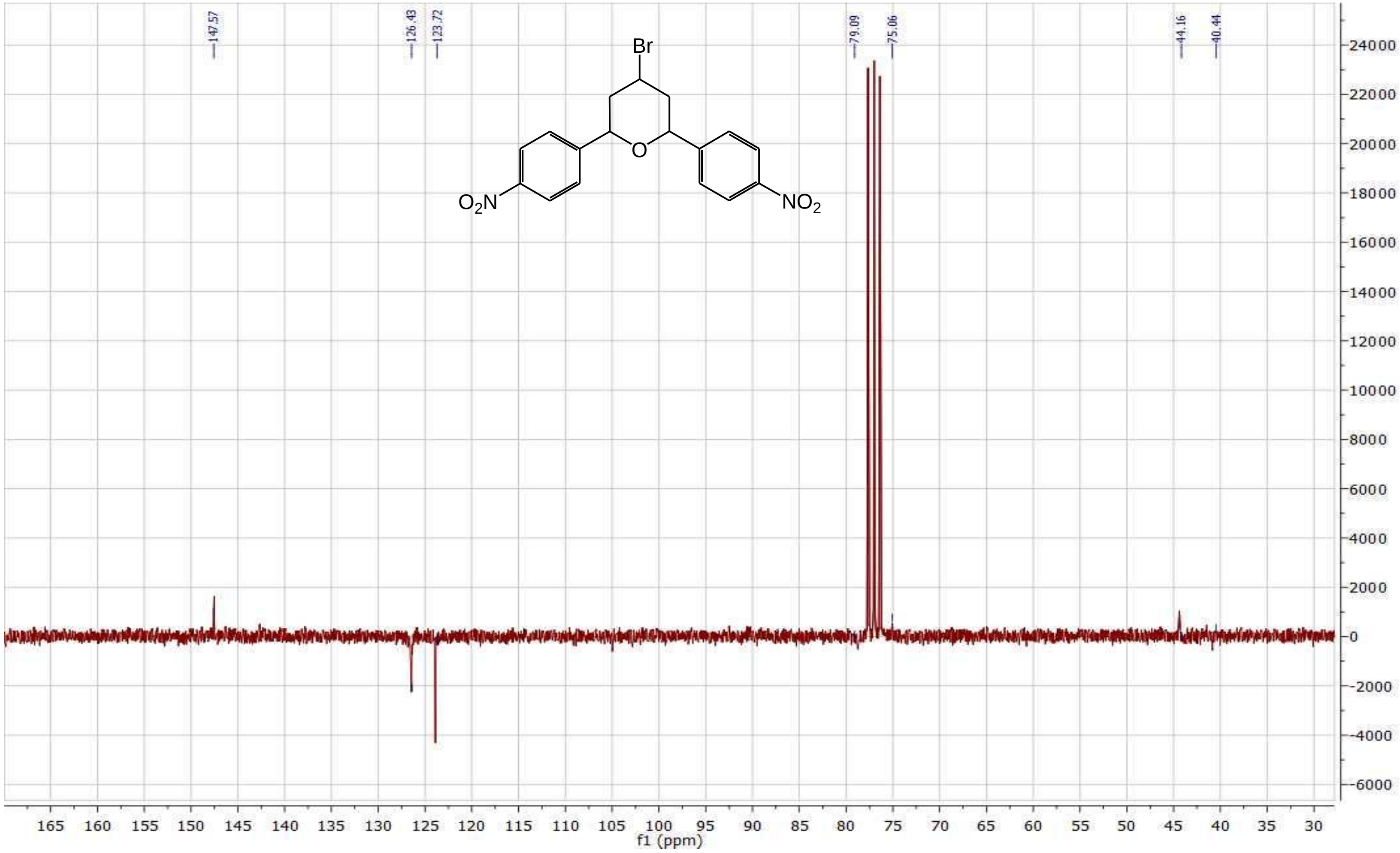

Espectro S10 - Espectro de RMN ^{13}C ($CDCl_3$, 125 MHz) do 4-bromo-tetrahydro-2,6-bis(4-nitrophenyl)-2H-pyran (8b)

4-bromo-tetrahidro-2,6-dihexyltetrahidropyrano (9b): ^{1}H NMR (500 MHz, $CDCl_3$): δ = 4.12 (m, H_4 ax), 3.21 (m, H_2 ax e H_6 ax), 2.19 (dd, J = 10.0 Hz, H_3 eq e H_5 eq), 1.61 (m, H_3 ax e H_5 ax), 1.519 (m, 24H, $(CH_2)_6$), 0.829 (t, 6H, CH_3). ^{13}C NMR (125 MHz, $CDCl_3$): δ= 47.33, 43.43, 35.74, 31.65, 29.03, 25.34, 22.45, 13.94.

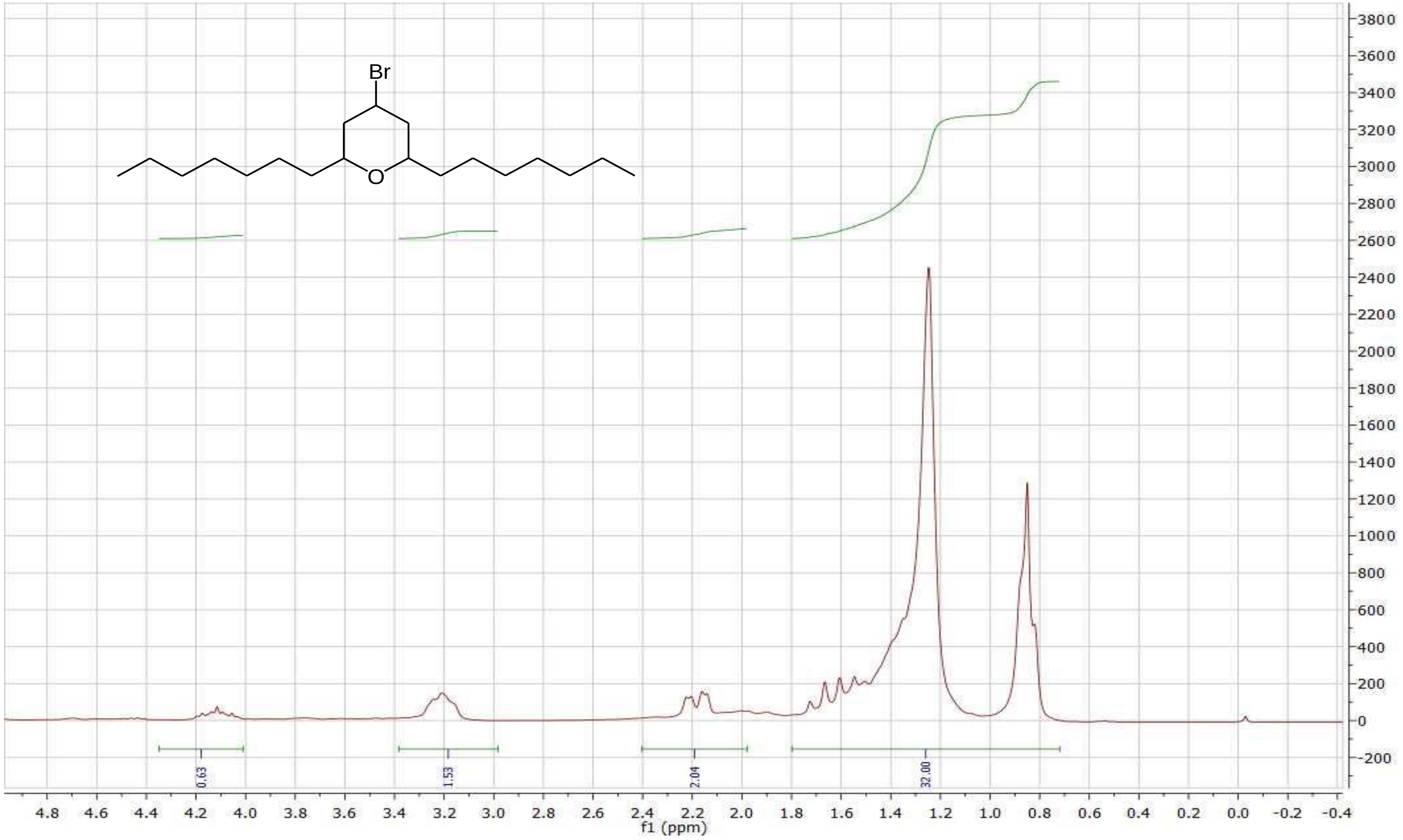

Espectro S11 - Espectro de RMN ^{1}H ($CDCl_3$, 500 MHz) do 4-bromo-2,6-diheptyl-tetrahydro-2H-pyran (9b)

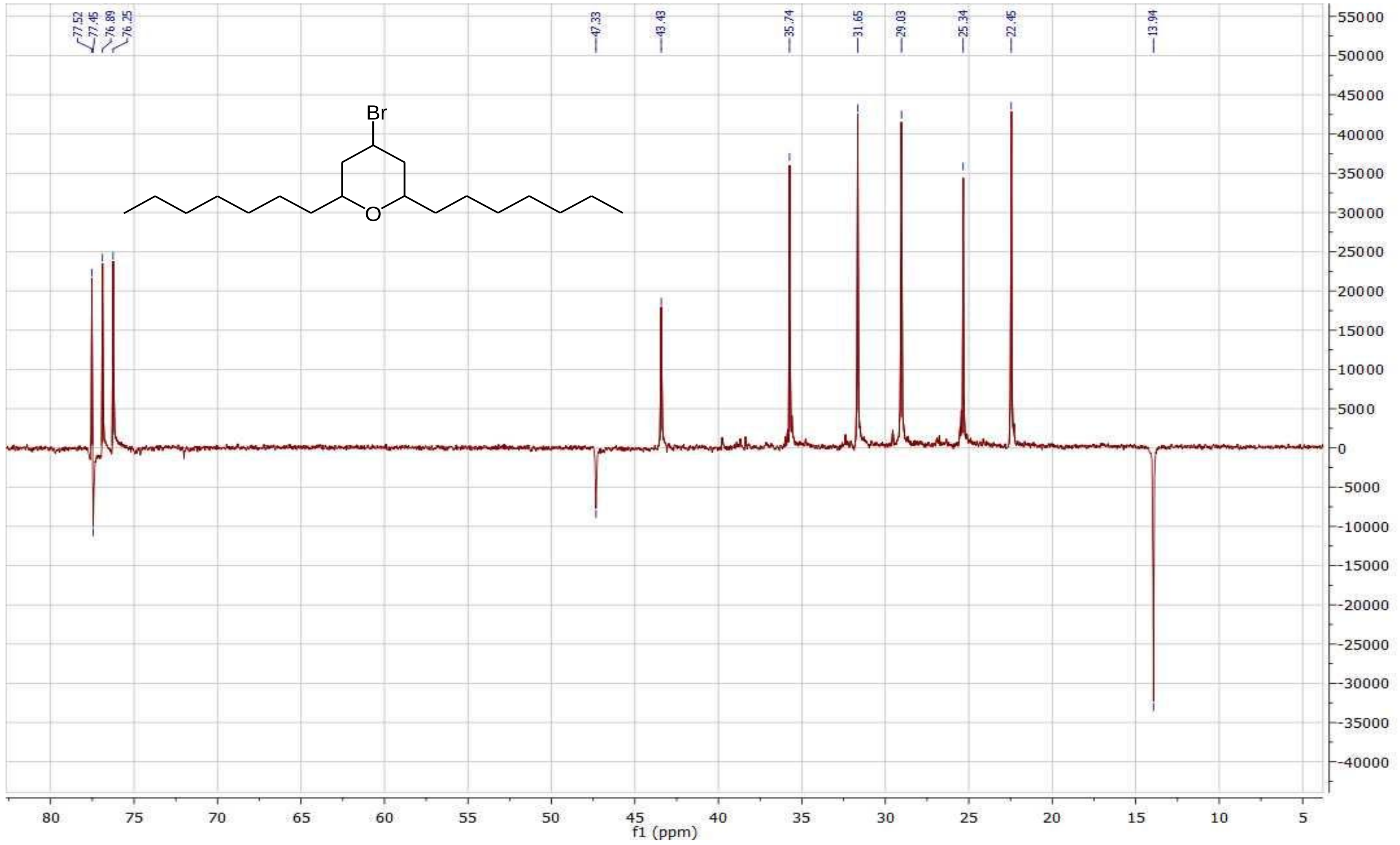

Espectro S12 - Espectro 10 - Espectro de RMN ^{13}C ($CDCl_3$, 125 MHz) do 4-bromo-2,6-diheptyl-tetrahydro-2H-pyran (9b)
